# Faroe Islands rephotography image registration dataset

**DOI:** 10.1016/j.dib.2023.108979

**Published:** 2023-02-13

**Authors:** Axel Schaffland, Julius Schöning

**Affiliations:** a Osnabrück University, Postfach 44 69, Osnabrück 49069, Germany; b Osnabrück University of Applied Sciences, Albrechtstr. 30, Osnabrück 49076, Germany

**Keywords:** Rephotography, Then and now, Faroes, Landscape change, Cultural heritage, Multitemporal image registration

## Abstract

Over 200 georeferenced registered rephotographic compilations of the Faroe Islands are provided in this dataset. The position of each compilation is georeferenced and thus locatable on a map. Each compilation consists of a historical and a corresponding contemporary image showing the same scene. With steady object features, these two images of the same geolocation are aligned pixel accurately. In the summer of 2022, all contemporary images were photographed by A. Schaffland, while historical images were retrieved from the National Museum of Denmark collections.

Images show Faroese landscape and cultural heritage sites, focusing on relevant areas when the historical images were taken, e.g., Kirkjubøur, Tórshavn, and Saksun. Historic images date from the end of the 19th century to the middle of the 20th century. The historical images were taken by scientists, surveyors, archaeologists, and painters.

All historical images are in the public domain, have no known rights, or are shared under a CC license. The contemporary images by A. Schaffland are released under CC BY-NC-SA 4.0.

The dataset is organized as a GIS project. Historic images, not already georeferenced, were referenced with street view services. All historical images were added to the GIS database, containing camera position, viewing direction, etc. Each compilation can be displayed as an arrow from the camera position along the view direction on a map. Contemporary images were registered to historical images using a specialized tool. None or only a suboptimal rephotograph could be taken for some historical images. These historical images are still added to the database together with all other original images, providing additional data for improvements in rephotography methods in the upcoming years.

The resulting image pairs can be used in image registration, landscape change, urban development, and cultural heritage research. Further, the database can be used for public engagement in heritage and as a benchmark for further rephotography and time-series projects.

**Specifications Table**

**Table:** 

Subject	Computer Vision and Pattern Recognition
Specific subject area	Registered and georeferenced image pairs consisting of a historical and contemporary image depicting the same scene of the Faroe Islands.
Type of data	Raw Image
How the data were acquired	Historic Images were acquired from https://samlinger.natmus.dk/. Contemporary images were taken with a Pentax KP with a Pentax HD DA 16-85mm f/3.5-5.6 ED DC WR lens.
Data format	Raw
Description of data collection	Historical images of the Faroe Islands were retrieved from the Collections of the National Museum of Denmark and added if they were already georeferenced or could be referenced by A. Schaffland. Contemporary images were added if the camera position of the historic image could be reached. For some historic images, no or only inadequate rephotographs were taken.
Data source location	Country: Faroe Islands Latitude and longitude (and GPS coordinates, if possible) for collected samples/data: GPS coordinates are provided per compilation in the GPKG database
Data accessibility	Repository name: Faroe Islands Rephotography Image Registration Dataset [1] Data identification number: 10.26249/FK2/QBSVQJ Direct URL to data: 10.26249/FK2/QBSVQJ

## Value of the Data

Rephotographies are used widely in the scientific community. Webb et al. discuss rephotography for the natural sciences [2] and Schaffland et al. give a broader view on the applications of rephotography [3]. The rephotographic compilations of this dataset are of value to several divisions of the scientific community:

• **Computer Vision:** The data consist of image pairs of historic monochrome images and contemporary colour images. Since the images are registered, they can be used to develop, train, and evaluate (deep) multitemporal image registration, especially those geared towards historical images [4,5].
• **Landscape Change:** Image pairs depicting the Faroese landscape can be used to study and visualize the landscape and land usage change on the Faroe Islands. This is the origin of rephotography, confirmed by the large number of publications [6,7].
• **Cultural Heritage:** Images taken of heritage sites, e.g., Kirkjubøur and Dúvugarðar, track these sites over time, measure the decay, and observe restoration efforts, as previously done on other sites [8,9].
• **Urban Development:** Image pairs located in Tórshavn and other towns can be used to study and visualize urban development, as already done for other cities [10].
• **Public Heritage Engagement:** All image pairs can raise public interest in heritage. Rephotographs accompanying popular science publications serve as a link to the past, allowing us to make connections between then and now, also in an educational environment [11].
• **Future Rephotography and Time Series:** Since all image pairs are georeferenced with camera position and view direction, they can also serve as a benchmark for future rephotography and the creation of time series as previously done for other regions [12,13]. For future projects, the preparation time is significantly reduced since manually georeferencing the images is already completed.

## 1 Objective

Datasets of registered image pairs are required to develop, train and evaluate (deep) multitemporal multimodal image registration algorithms. This dataset consists of registered image pairs featuring historic monochrome images taken with different cameras at different points in time, and contemporary colour rephotographs taken with a different camera. These registration algorithms could be used to (semi)automatical register rephotographs, but also other image pairs taken at different points in time with different cameras.

Further, the georeference rephotographs allow us to observe the landscape and land usage change, observe urban development and track cultural heritage on the Faroe Islands. Up till now, no publicly available collections of Rephotographs of the Faroe Islands exist.

## 2 Data Description

The raw data is available under 10.26249/FK2/QBSVQJ.

The raw data consists of the following parts:

• **faroeRephotos.qgz:** The QGIS file for this project. More information can be found in the `README.md`.
• **faroeRephotos.gpkg:** The project database containing the metadata of the images, including location, comment, title, authors, and copyright.
• **README.md:** A text file containing the data description and further information.
• **registered:** A directory with the registered image pairs with the following naming scheme: *<old name>_registered.<extension>, <old name>_<new name>_registered.<extension>*, respectively. The names can be retrieved from the database. Images can also be displayed inside QGIS.
• **unregistered:** A directory with the unregistered images, with the following naming scheme: *<old_name>.<extension>, <old_name>_<new_name>.<extension>,* respectively*.* Not for all old images; new images exist. The names can be retrieved from the database. Images can also be displayed inside QGIS.

## 3 Experimental Design, Materials and Methods

**Fig. 1:**
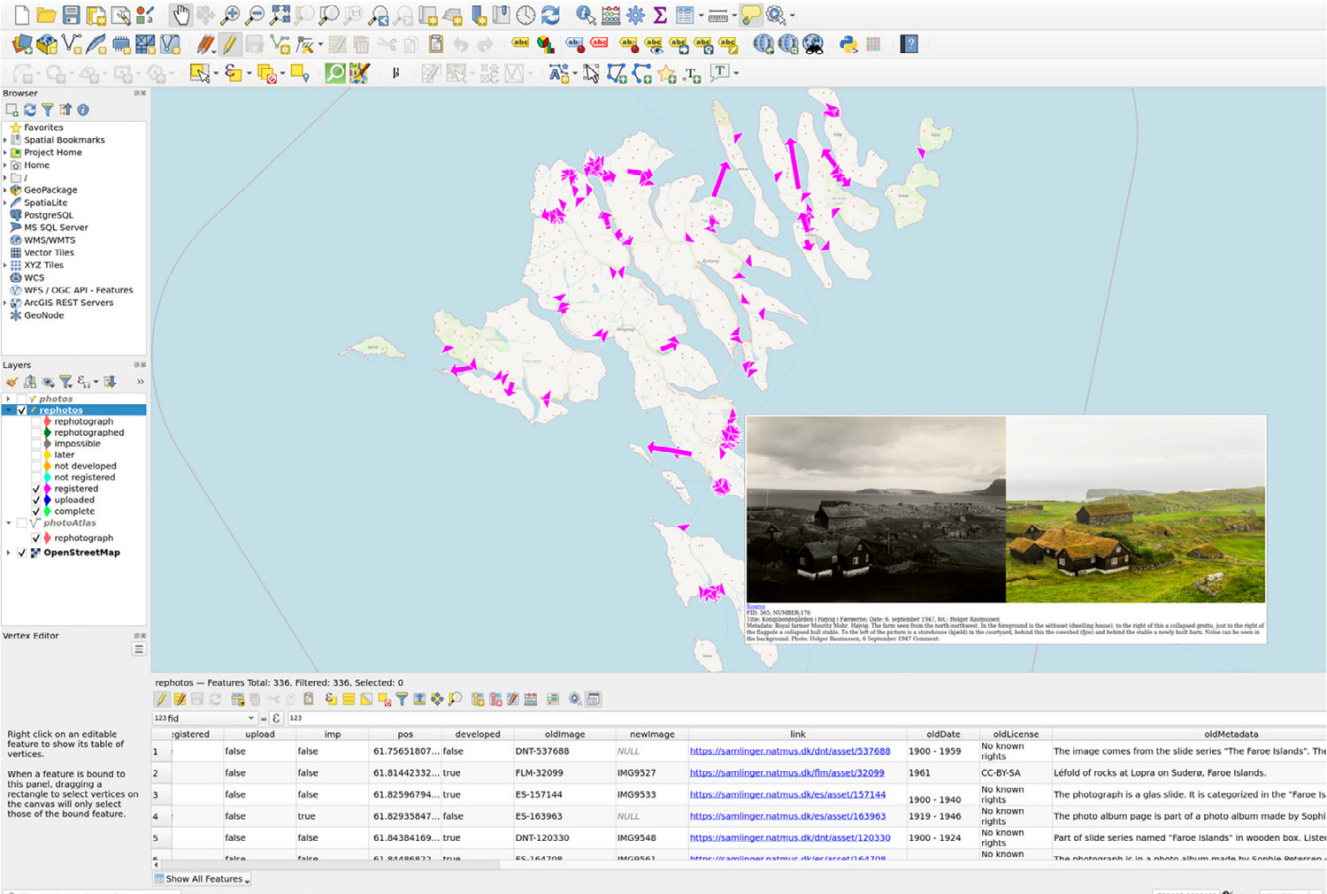
Screenshot of the QGIS project with an example compilation displayed.

Historic images were acquired from the online collections of the National Museum of Denmark [14] using *Færøerne* or *Føroyar* as keywords. Note that the metada is mainly in Danish, with few fractions in Faroese. Images without georeference and no possibility to determine it were excluded, e.g., portraits and featureless landscapes. Some of the images were already georeferenced. For the other images, metadata and Google Street View was used for georeferencing. Georeferenced images were added to the database. QGIS, together with OpenStreetMap data, was used for the management of the database and organization of the project. The database contains one entry/feature for each compilation with names, camera position, viewing direction, source link, author, license, date information, and various progress fields. As shown in Fig. 1, each compilation is displayed as an arrow on a map in QGIS. The arrows start from the camera position and point in the view direction. For use in the field, an atlas was printed containing all images together with a map section of the surrounding area.

On the Faroe Islands, the locations were visited over two weeks in the Summer of 2022. Printed historical images were compared with the real world until the correct position was found using classic analog rephotography methods [3]. Then a contemporary image was taken and verified. Contemporary images were taken with a Pentax KP with a Pentax HD DA 16-85mm f/3.5-5.6 ED DC WR lens. Progress was tracked in the Atlas and QGIS, depending on weather and accessibility.

After data collection, digital negative processing of the contemporary images was done with Adobe Photoshop 2021/22. The processed images were registered to the historical images with a specialized program [15], keeping the historic images fixed and transforming the contemporary image with a rigid transformation matrix.

## Ethics Statements

This study did neither involve human subjects and animal experiments nor was data collected from social media platforms. The authors confirm that the historical images were retrieved in compliance with the data redistribution policy of the online platform.

## CRediT authorship contribution statement

**Axel Schaffland:** Conceptualization, Methodology, Software, Investigation, Data curation, Writing – original draft, Writing – review & editing. **Julius Schöning:** Writing – review & editing, Supervision.

## Declaration of Competing Interest

The authors declare that they have no known competing financial interests or personal relationships that could have appeared to influence the work reported in this paper.

## Data Availability

Faroe Islands Rephotography Image Registration Dataset (Original data) (osnaData).
